# Validation of the Burden Transfer Inventory‐abbreviated and examination across veterinary medicine positions and settings in the United States

**DOI:** 10.1002/vro2.46

**Published:** 2022-10-28

**Authors:** Mary B. Spitznagel, John T. Martin, Mark D. Carlson, Christopher M. Fulkerson

**Affiliations:** ^1^ Department of Psychological Sciences Kent State University Kent Ohio USA; ^2^ Stow Kent Animal Hospital Kent Ohio USA; ^3^ Department of Veterinary Clinical Sciences College of Veterinary Medicine and Center for Cancer Research Purdue University West Lafayette Indiana USA

## Abstract

**Background:**

Burden transfer, when veterinary client caregiver burden underlies stressful encounters with providers, elevates risk for occupational distress in veterinary medicine. To date, burden transfer has been primarily examined in veterinarians working in general practice, using methods that are time consuming. The current work validates an abbreviated Burden Transfer Inventory (BTI‐A) and explores burden transfer across positions of employment and veterinary settings.

**Methods:**

Participants completed online measures of burden transfer, stress and burnout. A BTI‐A with items representing each BTI domain was created with an initial validation sample (*n* = 1151 veterinarians). Confirmatory psychometric analyses were conducted in a cross‐validation sample (*n* = 440 veterinarians and support staff), followed by exploration of the BTI and BTI‐A across veterinary settings and position of employment.

**Results:**

The BTI‐A correlated with the full‐length BTI (*r* = 0.89–0.96) shows good internal consistency (*α* = 0.72–0.88) and 1‐month test–retest reliability (*r* = 0.69–0.74). The BTI‐A correlated significantly (*p* < 0.001) with stress and burnout. Exploratory comparisons suggested group differences including greater reactivity in general compared to specialty referral/emergency practice (*p* = 0.02).

**Conclusion:**

The BTI‐A can be used in place of the original measure when brevity is important. Use of the BTI‐A may help guide allied mental health professionals in providing support for wellbeing in veterinary healthcare team members.

## INTRODUCTION

Prior work suggests that ‘burden transfer’ is a key source of stress for veterinary medicine personnel.^1^ The concept of burden transfer suggests that caregiver burden in the veterinary client leads to stressful encounters with the provider, shifting burden to veterinary personnel.^2^ Five domains of such veterinarian–client interactions have been identified via factor analysis: (1) daily hassles (examples are clients requesting impossible predictions, showing poor memory or comprehension), (2) affect (examples are clients exhibiting grief or sadness, needing euthanasia counselling), (3) non‐adherent/inconsiderate behaviours (examples may include clients declining investigation or treatment recommendations), (4) confrontations (examples may include clients refusing to pay for services, blaming for a negative outcome) and (5) excess communications (examples may include surfeit of phone or email contact). Interactions within these domains strongly relate to caregiver burden in veterinary clients and significantly predict stress and burnout for veterinarians.^1^

Although the frequency of such interactions predicts the negative outcomes, the mere presence of these situations is not nearly as predictive as the veterinary provider's *reaction* to these interactions.^1^ A potentially modifiable risk factor for occupational distress in the field is thus identified. Pilot work has demonstrated that targeting reactivity to difficult client interactions with an educational programme can lead to significantly reduced burden transfer and promising decreases in stress and burnout.^2^ These findings emphasise the importance of efficient and reliable measurement of burden transfer.

To date, one assessment of burden transfer has been conducted using the Burden Transfer Inventory (BTI).^1^ However, with 33 items asking about both frequency and reaction, this measure may be cumbersome to include in research or assessment of busy professionals. Moreover, the instrument measuring this construct was developed in a sample of veterinarians, largely working in general practice. Accordingly, the goal of the present study was to: (1) reduce BTI items to a psychometrically sound abbreviated version, (2) cross‐validate an abbreviated BTI (BTI‐A) in broader samples of veterinary professionals and (3) explore the BTI and BTI‐A across various veterinary settings and categories of employment.

## MATERIALS AND METHODS

### Participants

The present work drew upon data from two sources. The original BTI normative sample^1^ was used as an initial validation group to create a new abbreviated measure with psychometric properties warranting further development. New data were collected for the cross‐validation sample. For both samples, participants were required to provide complete data, to have current employment involving client interaction in clinical veterinary practice and the ability to comprehend/respond to measures in English.

The initial validation sample group has been previously described^1^ and was a sample of veterinarians recruited through the Veterinary Information Network. A mass email message was sent to 37,305 veterinarians. Of 1170 responses received, 19 were excluded due to working in a setting without client contact, leaving a total of 1151 (3.1% of the initial reach) responses.

The cross‐validation sample group involved representation from a variety of settings (including general practice, specialty referral/emergency medicine and academic veterinary medical centre) and positions of employment (including veterinarian, registered veterinary technician/nurse, non‐certified technician/assistant, customer service representative and management). Participants in this sample were enrolled for a 3‐h continuing education programme about burden transfer and consented to research. A subset of these participants (*n* = 70) provided 1‐month retest data. Only measures collected prior to the programme were included in the dataset for the current study. From 443 participants, three were excluded for working in a position that was not client‐facing, giving a sample of 440 individuals.

### Measures

#### The Burden Transfer Inventory

The BTI^1^ is a validated instrument developed to assess burden transfer in veterinary medicine. The respondent is asked to endorse both Frequency of and Reaction to the five domains of client interaction as given above, namely daily hassles, affect, non‐adherent/inconsiderate, confrontation and excess communication. The original BTI included 33 items across these domains. Each item is rated for Frequency to indicate how often the client interaction occurs (0 = never occurred, 1 = has occurred, but not in past week, 2 = once or twice in past week, 3 = approximately daily, 4 = more often than daily) and Reaction to indicate how bothersome the situation is (0 = not at all, 1 = only a little, 2 = moderately, 3 = quite a lot, 4 = extremely). Items are summed for subscales of Frequency and Reaction, with higher Frequency scores indicating more encounters of this nature; with higher Reaction scores indicating higher reactivity to these situations.

#### The Perceived Stress Scale

The Perceived Stress Scale (PSS)^3^ is a previously validated measure of stress perception, containing items asking about current level of stress or feelings that life is unpredictable or overloaded. This 10‐item scale rates each item using a Likert‐type response format (0 = never, 1 = almost never, 2 = sometimes, 3 = fairly often, 4 = very often), which are summed with reverse scoring (namely, 0 = very often, 1 = fairly often, 2 = sometimes, 3 = almost never, 4 = never) as indicated for specified items. A higher score indicates greater stress.

#### The Copenhagen Burnout Inventory

The Copenhagen Burnout Inventory (CBI)^4^ is a previously validated measure of burnout. Due to the psychological nature of this measure and complexity of scoring, it is strongly recommended that individuals intending to use the CBI refer to original sources, rather than unverified online sources. The CBI comprises three subscales: personal, work‐related and client‐related burnout. Items utilise a five‐point response format (0 = never/almost never, 1 = seldom, 2 = sometimes, 3 = often, 4 = always or 0 = to a very low degree, 1 = to a low degree, 2 = somewhat, 3 = to a high degree, 4 = to a very high degree) and are summed for each subscale. Higher scores indicate greater burnout in that domain.

#### Additional data

Participants self‐reported demographic information include age, ethnicity, gender, nature of employment and practice setting, together with length of time in the profession.

### Procedure

Initial validation data were extracted from the original BTI dataset (see Supporting Information S1)^1^; new data for cross‐validation were collected between January 2020 and November 2021. To abbreviate the BTI, previously reported^1^ correlation coefficients between BTI item and the BTI domain to which it was assigned were examined for contribution to the scale. To maintain equal subscale representation while minimising the overall number of items on the BTI‐A, the two items from each domain with the strongest correlation were selected (correlation ranged from 0.41 to 0.73; see Table 1 for selected items). Examination of psychometric properties was then undertaken, including investigation of internal consistency (including degree to which items align in reliably measuring a construct), construct validity (including degree to which an overall measure correlated with other measures of the same construct or related construct) and 1‐month test–retest reliability (stability of the measure over a period of time). Analyses were conducted as described below.

**TABLE 1:** Outline of the Burden Transfer Inventory (BTI)‐Abbreviated

	Frequency response format	Reaction response format
	0 = Never occurred	0 = Not at all
	1 = Has occurred, but not in past week	1 = A little
	2 = Once or twice in the past week	2 = Moderately
	3 = Approximately daily	3 = Very much
	4 = More often than daily	4 = Extremely
	N/A = Does not apply to my work environment	N/A = Has not occurred

	Frequency	Reaction
1. Client shows poor memory or comprehension for instructions (D)	0 1 2 3 4 N/A	0 1 2 3 4 N/A
2. Client conducts ‘research’ (e.g., online searches) about pet's problems or disease (D)	0 1 2 3 4 N/A	0 1 2 3 4 N/A
3. Client demonstrates grief or sadness (A)	0 1 2 3 4 N/A	0 1 2 3 4 N/A
4. Client requires euthanasia counselling (A)	0 1 2 3 4 N/A	0 1 2 3 4 N/A
5. Client declines recommended work‐up (N)	0 1 2 3 4 N/A	0 1 2 3 4 N/A
6. Client declines recommended treatment (N)	0 1 2 3 4 N/A	0 1 2 3 4 N/A
7. Client unwilling to pay (C)	0 1 2 3 4 N/A	0 1 2 3 4 N/A
8. Client blames you for poor outcomes (e.g., failure to improve, death) (C)	0 1 2 3 4 N/A	0 1 2 3 4 N/A
9. Repeated or unsolicited client email contact (E)	0 1 2 3 4 N/A	0 1 2 3 4 N/A
10. Repeated or unsolicited client telephone contact (E)	0 1 2 3 4 N/A	0 1 2 3 4 N/A
**SCORING**—Sum responses for each column (Frequency and Reaction). Write the total score for each below.		

Subscale totals	Frequency (your score)	Reaction (your score)
Total score		

*Note*: Letters in parentheses following each item indicate subscale from the original BTI: (D) refers to daily hassles, (A) refers to affect, (N) refers to non‐adherence/inconsiderate behaviour, (C) refers to confrontations and (E) refers to excess communication.

Below is a list of client‐related situations that people working in veterinary medicine sometimes encounter. Using the below scales, please first indicate *how often these have occurred for you* (‘Frequency’). Then, indicate *how much the situations have bothered or upset you* (‘Reaction’).

### Statistical analyses

Statistical analyses were conducted using SPSS version 28.0 (IBM, Armkonk, NY, USA). First, demographic information was characterised for both samples using descriptive statistics. All variables used in statistical analyses were evaluated for normality using histograms and skewness/kurtosis to ensure parametric assumptions were met.

For the initial validation sample, the BTI‐A was compared to the full‐length BTI using Pearson bivariate correlation. The BTI‐A was then examined for internal consistency using Cronbach's alpha. Relationships between the BTI‐A and the PSS and CBI were examined via Pearson bivariate correlations for evidence of construct validity.

Analyses were repeated for the BTI‐A in the cross‐validation sample. Because no prior work has examined the full‐length BTI in a sample with mixed representation (i.e., multiple settings and positions of employment), these analyses were conducted for the full‐length version, as well. In addition, test–retest reliability using Pearson bivariate correlation was examined for both measures in the sample subset with 1‐month retest data.

The full‐length BTI and BTI‐A were then examined in the cross‐validation sample using analysis of variance. Comparisons were examined for: (1) position of employment (including veterinarian, registered veterinary technician/nurse, non‐certified technician/assistant, customer service representative and management) and (2) categories of veterinary setting (including general practice, specialty referral/emergency and academic medical centre). The familywise alpha level was set at 0.05, with Bonferroni post hoc follow‐up as appropriate.

## RESULTS

### Initial validation

For sample demographics and measure descriptive statistics, see Table 2. Participants, all employed as veterinarians, comprised primarily of females and identified as White, averaging approximately 46 years of age and 18 years working in the field. Correlation of the BTI‐A with the original full‐length BTI was *r* = 0.93 for Frequency and *r* = 0.89 for Reaction. Internal consistency for the BTI‐A was *α* = 0.82 for Frequency and *α* = 0.72 for Reaction. The BTI‐A showed highly significant correlations with the PSS and CBI (all *p* < 0.001; see Table 3).

**TABLE 2:** Demographic characteristics and descriptive statistics for initial and cross‐validation samples

	Initial validation (*N* = 1151)	Cross‐validation (*N* = 440)
**Demographic characteristics**		
Age (M/SD)	45.83/11.52	37.28/10.89
Years in the field (M/SD)	18.12/11.47	11.63/9.04
Gender (*N*, %)		
Female		
Male	931, 80.9%	400, 90.9%
Other or prefer not to say	214, 18.6%	38, 8.6%
Ethnicity (*N*, %)		
White or Caucasian	1044, 90.7%	401, 91.1%
Asian American or Pacific Islander	31, 2.7%	5, 1.1%
Latin American or Hispanic	19, 1.7%	22, 5.0%
African American or Black	4, <1%	3, <1%
Native American or indigenous	–	3, <1%
Other or prefer not to say	35, 3.0%	6, 1.4%
Setting (*N*, %)		
General practice	975, 84.7%	175, 39.8%
Specialty referral/emergency	155, 13.5%	235, 53.4%
Other (includes academic)	21, 1.8%	30, 6.8%
Position of employment (*N*, %)		
Veterinarian	1151, 100%	99, 22.5%
Certified veterinary technician/nurse	–	99, 22.5%
Veterinary assistant (unlicensed)	–	86, 19.5%
Customer service representative	–	70, 15.9%
Management	–	70, 15.9%
Other (e.g., social media, social worker)	–	16, 3.6%
**Measure descriptive statistics (Mean/Standard Deviation; min–max)**		
Burden Transfer Inventory		
Total Frequency	62.94/16.70; 0–131	59.54/23.62; 0–127
Total Reaction	35.66/18.19; 0–99	50.30/25.07; 0–127
Burden Transfer Inventory‐Abbreviated		
Total Frequency	20.78/5.63; 0–40	18.19/7.85; 0–40
Total Reaction	10.79/5.48; 0–32	14.13/7.43; 0–40
Perceived Stress Scale	18.05/6.54; 0–36	19.61/5.71; 3–34
Copenhagen Burnout Inventory		
Personal burnout	313.05/123.97; 0–600	350.40/118.65; 25–600
Work‐related burnout	348.99/126.58; 0–625	378.35/141.08; 25–700
Client‐related burnout	275.83/146.41; 0–600	268.98/139.33; 0–600

**TABLE 3:** Correlations of stress and burnout with the Burden Transfer Inventory (BTI) and BTI‐Abbreviated (BTI‐A) in the initial and cross‐validation samples

	Perceived Stress Scale	Copenhagen Burnout Inventory		
	Total	Personal	Work	Client
Initial validation sample				
BTI‐A total Frequency	0.19	0.24	0.28	0.30
BTI‐A total Reaction	0.41	0.41	0.44	0.48
Cross‐validation sample				
BTI total Frequency	0.28	0.34	0.38	0.39
BTI total Reaction	0.41	0.40	0.46	0.50
BTI‐A total Frequency	0.25	0.33	0.38	0.38
BTI‐A total Reaction	0.40	0.42	0.47	0.47

*Note*: All correlations were significant at *p* < 0.001.

### Cross‐validation

For sample demographics and measure descriptive statistics, see Table 2. Participants were primarily females identifying as White. Average age was approximately 37 years with just under 12 years working in the field. Similar distribution across positions of employment was observed and a majority worked in specialty referral/emergency settings. Comparison of primary measures showed lower BTI, BTI‐A, PSS and CBI personal and work‐related burnout (all *p* ≤ 0.001) in initial validation versus cross‐validation samples.

In the cross‐validation sample, internal consistency for the full‐length BTI was *α* = 0.96 for both Frequency and Reaction. One‐month test–retest reliability was *r* = 0.82 for Frequency and *r* = 0.78 for Reaction. Correlations with the PSS and subscales of the CBI were greater for Reaction than for Frequency; all were highly significant (*p* < 0.001; see Table 3).

Correlation of the BTI‐A with the original full‐length BTI was *r* = 0.96 for Frequency and *r* = 0.95 for Reaction. Internal consistency was *α* = 0.88 for Frequency and *α* = 0.85 for Reaction. One‐month test–retest reliability was *r* = 0.74 for Frequency and *r* = 0.69 for Reaction. The BTI‐A showed a pattern of correlations with the PSS and CBI similar to the full‐length version; again, all correlations were highly significant (*p* < 0.001; see Table 3).

### Exploration of position and setting

Examining position of employment, significant differences neither emerged on full‐length BTI Frequency [*F*(4, 419) = 0.48, *p* = 0.75] or Reaction [*F*(4, 419) = 1.90, *p* = 0.11] subscales, nor on BTI‐A Frequency [*F*(4, 419) = 0.44, *p* = 0.78] or Reaction [*F*(4, 419) = 1.90, *p* = 0.11]. Comparisons of veterinary setting also yielded no significant differences for Frequency on the BTI [*F*(2, 438) = 0.35, *p* = 0.55] or BTI‐A [*F*(2, 438) = 0.05, *p* = 0.83]. Reaction did not significantly differ for the BTI‐A [*F*(2, 438) = 2.42, *p* = 0.09], but it did for the full‐length BTI [*F*(2, 438) = 4.07, *p* = 0.02]. Post hoc Bonferroni follow‐up indicated that individuals working in general practice showed greater Reaction compared to those in specialty referral/emergency settings (*p* = 0.02). See Table 4 for group descriptive statistics.

**TABLE 4:** Comparison across position of employment and veterinary setting for Burden Transfer Inventory (BTI) and BTI‐Abbreviated (BTI‐A) in the cross‐validation sample

	BTI Frequency	BTI Reaction	BTI‐A Frequency	BTI‐A Reaction
Position of employment (M/SD; min–max)				
Veterinarian (*n* = 99)	60.83/19.97; 0–117	55.58/22.72; 0–104	19.22/6.63; 0–36	15.24/6.66; 0–40
Certified technician/nurse (*n* = 99)	59.81/23.73; 0–124	53.18/25.12; 0–108	18.27/7.90; 0–40	15.53/7.52; 0–31
Veterinary assistant (non‐certified) (*n* = 86)	61.07/22.92; 0–127	52.00/27.49; 0–127	18.21/7.29; 0–40	14.48/7.94; 0–36
Customer service representative (*n* = 70)	63.93/21.23; 29–124	46.51/21.56; 0–113	19.10/7.85; 4–38	13.24/6.99; 0–35
Management (*n* = 70)	59.13/24.77; 0–113	48.20/23.02; 0–88	18.04/8.30; 0–33	13.17/6.71; 0–40
Veterinary setting (M/SD; min–max)^a^				
General practice (*n* = 174)	60.15/19.01; 0–113	54.47/21.34; 0–116	18.01/6.33; 0–34	15.07/6.54; 0–36
Specialty referral/emergency (*n* = 236)	59.82/26.50; 0–127	47.50/27.17; 0–127	18.50/8.74; 0–40	13.55/8.01; 0–40
Academic medical (*n* = 29)	54.41/23.75; 2–116	47.76/25.76; 2–104	17.00/8.47; 0–40	13.07/7.03; 0–27

Abbreviations: M, mean; SD, standard deviation.

^a^ Significant difference in veterinary setting, with general practice showing higher full‐length BTI Reaction compared to specialty referral/emergency (*p* = 0.02).

## DISCUSSION

The present study developed and examined psychometric properties of the BTI‐A and explored both measures across positions of employment and veterinary settings to understand potential differences in burden transfer. The abbreviated measure demonstrated strong correlations with the full‐length BTI and strong internal consistency^5^ across an initial validation sample of veterinarians and cross‐validation sample of individuals representing various positions and settings. Highly significant correlations were demonstrated between the BTI‐A and established measures of stress and burnout, providing support for construct validity. One‐month test–retest was more reliable for the full‐length BTI than BTI‐A, while the abbreviated format still showed good to excellent reliability.^6^

The BTI‐A may facilitate understanding of burden transfer in daily life for individuals working in veterinary medicine. Compared to the original measure, which is time consuming to complete and complicated to score, this brief assessment tool may be more easily incorporated into research or intervention programmes. In contrast to the full‐length BTI, which provides in‐depth examination of individual domains of burden transfer interaction, the BTI‐A produces overall Frequency and Reaction scores only. The full‐length BTI may thus still be optimal to use in cases where it is desirable to identify specific domains of burden transfer in which a person struggles most. However, the brevity of administration and ease of scoring likely make the BTI‐A preferable in screening and educational situations. Because published studies suggest that targeting reactivity to difficult client interactions can reduce burden transfer, and potentially stress and burnout in turn,^2^ allied mental health providers (such as veterinary social workers) could use this tool to work with team members to help identify reactivity around client interactions, examining change before and after intervention. In this way, measuring and targeting burden transfer reactivity may benefit mental health and wellbeing in the field.

Two factors that might impact responses on the abbreviated or full‐length BTI, position of employment and veterinary setting, were explored in the current work. Although position of employment did not reveal significant differences among the groups examined, both versions of the BTI showed a trend towards a difference in the overall Reaction subscale. Future work should determine if group differences in a specific burden transfer domain underlie this trend. For example, it is possible that clinical staff would show greater reactivity to excess communication compared to customer support staff, as handling client contact is a primary description of that role, making frequent telephone or email contact an expected part of the position. Similarly, comparisons of veterinary settings suggested that individuals working in general practice experience greater reactivity compared to specialty referral/emergency settings. Although tempting to interpret this to different workplace demands or clientele (including greater financial resources in specialty clients compared to general practice^7^ might lead to lower non‐adherence or confrontations), these explanations would seemingly drive lower Frequency as well as Reaction. Perhaps some self‐selection is present, with individuals who better tolerate stressful interpersonal interactions being more likely to choose to work in the specialty referral/emergency setting. Notably, average responses from individuals in academic medical centres were similar to those from specialty referral/emergency settings; although the difference in the academic versus general practice comparison was non‐significant, this was likely due to the small size of the academic medical sample. Overall, relationships between burden transfer and both veterinary setting and position of employment warrant further attention.

When developing a new measure, it is optimal to provide normative data.^8^ In preparing the current work, values for the full‐length BTI Reaction were very obviously lower in the initial validation compared to the cross‐validation sample. Differences could relate to varied settings or positions as discussed above. Alternatively, there could be an influence of individual characteristics such as age or gender. The current study was neither designed nor powered to examine these questions, but these should be considered in future work. Timing and the influence of the global pandemic also warrant consideration. All initial validation data were collected prior to the pandemic, while most cross‐validation data were collected during this time. Greater daily life strain associated with the pandemic may leave veterinary personnel with fewer emotional resources to cope with difficult client interactions. Indeed, higher levels of not only burden transfer, but also stress and burnout were observed in the cross‐validation relative to the initial validation sample. As such, while psychometrics including reliability and validity of the BTI‐A can be established, it is prudent to wait until the world returns to greater normalcy to establish normative values associated with the BTI‐A. The current work lays the foundation for that next step. Regardless, the similarity of psychometric properties observed across the two samples (in spite of ostensible sample differences) suggests the measure is robust to heterogeneity of this nature.

Limitations of the current work were identified and may point to future directions. With over 90% of the sample self‐identifying as White, lack of diversity is important to note. Under‐representation of ethnic and racial minorities is well‐known to exist in the field of veterinary medicine in the United States.^9^ Because this work was undertaken in samples of primarily White individuals, it could represent the state of veterinary medicine but still not reflect the experience of those who are under‐represented. In developing normative values, focused efforts to include a greater diversity of individuals will be key. Additionally, given the potential impact of veterinary settings and positions of employment noted above, broad representation of these factors will be important for normative data, as well. Finally, due to observations that timing and the global pandemic may have influenced responses in the cross‐validation group, work to better understand the influence of the pandemic on burden transfer would be helpful, as well.

In conclusion, this work presents the BTI‐A, a rapid assessment tool to measure burden transfer in veterinary personnel. Findings support the psychometric properties of this instrument, including strong correlation with the full‐length BTI and evidence of sufficient construct validity, internal consistency and test–retest reliability. Given exploratory findings suggesting differences by veterinary setting and position of employment, normative data should be developed from a fully representative sample, optimally after pandemic‐related stress in the field decreases. In the meantime, the BTI‐A can be incorporated into research of wellbeing and client interactions in veterinary medicine, as well as in the practice of allied mental health providers to guide and assess success of interventions with veterinary personnel in the field.

## AUTHOR CONTRIBUTIONS

The authors confirm contribution to the paper as follows—*Study conception and design*: Mary B. Spitznagel. *Data collection*: Mary B. Spitznagel, Mark D. Carlson and John T. Martin. *Analysis and interpretation of results*: Mary B. Spitznagel. *Draft manuscript preparation*: Mary B. Spitznagel, Mark D. Carlson, Christopher M. Fulkerson and John T. Martin. All authors reviewed the results and approved the final version of the manuscript.

## CONFLICTS OF INTEREST

Mary Spitznagel routinely presents scientific work related to burden transfer and has received honoraria for doing so. The other authors have not declared any conflicts of interest.

## ETHICS STATEMENT

Study protocols were approved by the Kent State University Institutional Review Board. The current work was conducted and reported in accordance with STROBE (Strengthening the Reporting of Observational Studies in Epidemiology) criteria for cross‐sectional studies.

## Supporting information

The Supporting Information is provided with the permission of the AVMA and has been published as Supporting Information in the following article. Spitznagel MB, Ben‐Porath YS, Rishniw M, Kogan LR, Carlson MD. Development and validation of a Burden Transfer Inventory for predicting veterinarian stress related to client behavior. J Am Vet Med Assoc. 2019;254(1):133–44. doi: 10.2460/javma.254.1.133.

## Data Availability

The data that support the findings of this study are available from the corresponding author (Mary B. Spitznagel), upon reasonable request.
