# Supplementary material for: Validation of the Burden Transfer Inventory‐abbreviated and examination across veterinary medicine positions and settings in the United States
Source: Vet Rec Open. 2022 Oct 28;9(1):e46. doi: 10.1002/vro2.46 (PMC9614379; doi:10.1002/vro2.46)
Supplement: Supplementary file 1 — The Supporting Information is provided with the permission of the AVMA and has been published as Supporting Information in the following article. Spitznagel MB, Ben‐Porath YS, Rishniw M, Kogan LR, Carlson MD. Development and validation of a Burden Transfer Inventory for predicting veterinarian stress related to client behavior. J Am Vet Med Assoc. 2019;254(1):133–44. doi: 10.2460/javma.254.1.133. [file VRO2-9-e46-s001.pdf]

The Supporting Information is provided with the permission of the AVMA and has been published as Supporting Information in the following article. Spitznagel MB, Ben-Porath YS, Rishniw M, Kogan LR, Carlson MD. Development and validation of a Burden Transfer Inventory for predicting veterinarian stress related to client behavior. J Am Vet Med Assoc. 2019;254(1):133-44. doi: 10.2460/javma.254.1.133.

Supplementary Appendix S1—BTI tool. ©2018 M. B. Spitznagel.

Instructions: Below is a list of client-related situations that veterinarians sometimes encounter. Using the below scales, please first indicate *how often these have occurred for you* ("Frequency"). Next, please indicate how much the situations have bothered or upset you ("Reaction").

Frequency Response Format:
0=Never occurred
1=Has occurred, but not in past week
2=Once or twice in the past week
3=Approximately daily
4=More often than daily
N/A=Does not apply to my work environment

Reaction Response Format:
0=Not at all
1=Only a little
2=Moderately
3=Quite a lot
4=Extremely
N/A=Has not occurred

	Frequency	Reaction	Scale
1. Client wants diagnosis without diagnostic testing	0 1 2 3 4 N/A	0 1 2 3 4 N/A	N
2. Client is angry about patient's health status (e.g., lack of improvement)	0 1 2 3 4 N/A	0 1 2 3 4 N/A	C
3. Client demonstrates grief or sadness	0 1 2 3 4 N/A	0 1 2 3 4 N/A	A
4. Client requests services be conducted free of charge	0 1 2 3 4 N/A	0 1 2 3 4 N/A	C
5. Client requires euthanasia counseling	0 1 2 3 4 N/A	0 1 2 3 4 N/A	A
6. Repeated or unsolicited client telephone contact	0 1 2 3 4 N/A	0 1 2 3 4 N/A	E
7. Client shows poor memory for instructions	0 1 2 3 4 N/A	0 1 2 3 4 N/A	D
8. Client listens to advice from others (e.g., breeder, pet disease support group)	0 1 2 3 4 N/A	0 1 2 3 4 N/A	D
9. Client wants cure when there is none	0 1 2 3 4 N/A	0 1 2 3 4 N/A	C
10. Client blames you for poor outcomes (e.g., failure to improve, death)	0 1 2 3 4 N/A	0 1 2 3 4 N/A	C
11. Client declines recommended treatment	0 1 2 3 4 N/A	0 1 2 3 4 N/A	N
12. Client demonstrates anxiety	0 1 2 3 4 N/A	0 1 2 3 4 N/A	A
13. Repeated or unsolicited client email contact	0 1 2 3 4 N/A	0 1 2 3 4 N/A	E
14. Client complains about cost	0 1 2 3 4 N/A	0 1 2 3 4 N/A	N
15. Client has poor comprehension for instructions	0 1 2 3 4 N/A	0 1 2 3 4 N/A	D
16. Client unable to pay	0 1 2 3 4 N/A	0 1 2 3 4 N/A	C
17. Client unwilling to pay	0 1 2 3 4 N/A	0 1 2 3 4 N/A	C
18. Client does not efficiently communicate needed information	0 1 2 3 4 N/A	0 1 2 3 4 N/A	D
19. Client conducts "research" (e.g., online searches) about pet's problems or disease	0 1 2 3 4 N/A	0 1 2 3 4 N/A	D
20. Client wants impossible prediction of outcomes (e.g., how long will my pet live?)	0 1 2 3 4 N/A	0 1 2 3 4 N/A	D
21. Client upset about wait time in lobby	0 1 2 3 4 N/A	0 1 2 3 4 N/A	C
22. Client declines recommended euthanasia	0 1 2 3 4 N/A	0 1 2 3 4 N/A	C
23. Client makes complaint about you	0 1 2 3 4 N/A	0 1 2 3 4 N/A	C
24. Client makes complaint about your workplace	0 1 2 3 4 N/A	0 1 2 3 4 N/A	C
25. Client requires repeated communication for information you have already relayed	0 1 2 3 4 N/A	0 1 2 3 4 N/A	D
26. Client "shops around" to compare cost with other providers	0 1 2 3 4 N/A	0 1 2 3 4 N/A	D
27. Client is slow to make decisions in the exam room	0 1 2 3 4 N/A	0 1 2 3 4 N/A	N
28. Client wants to discuss own problems (including how pet causes problems for them)	0 1 2 3 4 N/A	0 1 2 3 4 N/A	D
29. Client alters treatment plan (e.g., medications) without consulting you	0 1 2 3 4 N/A	0 1 2 3 4 N/A	D
30. Client wants to be seen immediately when no immediate appointment is available	0 1 2 3 4 N/A	0 1 2 3 4 N/A	N
31. Client declines recommended work-up	0 1 2 3 4 N/A	0 1 2 3 4 N/A	N
32. Client calls when appointment would be more appropriate	0 1 2 3 4 N/A	0 1 2 3 4 N/A	N
33. Client fails to show up for scheduled appointment	0 1 2 3 4 N/A	0 1 2 3 4 N/A	N
Other:			

Subscale Totals	Frequency	Reaction
D (Daily Hassles)		

A (Affect)		
N (Nonadherent/Inconsiderate)		
C (Confrontation)		
E (Excess Communication)		
Total		
